# Co-Morbidity, Ageing and Predicted Mortality in Antiretroviral Treated Australian Men: A Quantitative Analysis

**DOI:** 10.1371/journal.pone.0078403

**Published:** 2013-10-25

**Authors:** Luis Furuya-Kanamori, Mark D. Kelly, Samantha J. McKenzie

**Affiliations:** 1 The University of Queensland, School of Population Health, Queensland, Australia; 2 Brisbane Sexual Health and HIV Service, Queensland, Australia; Infectious Disease Service, United States of America

## Abstract

**Background:**

Life expectancy has increased in HIV-positive individuals receiving combination antiretroviral therapy (cART); however, they still experience increased mortality due to ageing-associated comorbidities compared with HIV-negative individuals.

**Methods:**

A retrospective study of 314 Queensland HIV-infected males on cART was conducted. The negative impact of ageing was assessed by estimating the probability of 5-year mortality; comparisons were made between an HIV-specific predictive tool (VACS index) and the Australian Bureau of Statistics (ABS) life-tables to examine potential differences attributed to HIV. The negative impact of ageing was also assessed by the prevalence of comorbidities. Associations between comorbidity and estimates of predicted mortality by regression analysis were assessed.

**Results:**

The mean predicted 5-year mortality rate was 6% using the VACS index compared with 2.1% using the ABS life-table (p<0.001). The proportion of patients at predicted high risk of mortality (>9%) using the VACS index or ABS life-table were 17% and 1.8% respectively. Comorbidities were also more prevalent in this cohort compared with rates of comorbidities in age-matched Australian men from the general population. Metabolic disease (38.2%) was the most prevalent comorbidity followed by renal (33.1%) and cardiovascular disease (23.9%). Multivariate analysis demonstrated that patients with a history of cardiovascular disease had a higher predicted risk of mortality (OR=1.69;95%CI:1.17-2.45) whereas ex-smokers had a lower predicted risk of mortality (OR=0.61;95%CI:0.41-0.92).

**Conclusions:**

Using the VACS Index there is an increased predicted risk of mortality in cART-treated HIV infected Australian men compared with age-matched men using the ABS data. This increased predicted mortality risk is associated with cardiovascular disease and the number of comorbidities per subject; which suggests that the VACS Index may discriminate between high and low predicted mortality risks in this population. However, until the VACS Index is validated in Australia this data may suggest the VACS Index overestimates predicted mortality risk in this country.

## Introduction

Since the introduction of combination antiretroviral therapy (cART) in 1996, mortality patterns have shifted dramatically from the 26 AIDS-defining conditions to non-HIV specific causes of death, referred to as serious non-AIDS events (SNAE). This has resulted in increased life expectancy and improved quality of life for individuals with HIV [1,2]. Although life expectancy has increased dramatically for HIV-positive individuals receiving cART, it is still less than that of their uninfected peers [3,4]. Australians with HIV have 3.5 to 5 times increased risk of mortality when compared with the general population [5,6]. However, these studies report mortality rates dating back to the earlier cART era, it is not known if this excess mortality persists in contemporary practice.

Cardiovascular, metabolic, renal and oncological disorders account for the majority of SNAE. These ageing-specific comorbid conditions were shown in earlier studies to occur at a younger age in individuals with HIV compared with HIV-negative individuals [7].. These studies may have been confounded by population skewing [8]. More recent studies have indicated that some comorbid conditions occur at similar ages in HIV infected and HIV uninfected cohorts but with an increased adjusted age incidence in persons with HIV [9,10]. Collectively these studies reflect the complex interplay between HIV, age and comorbidity.

Recognition of this early mortality and higher number of ageing-associated comorbid conditions has led to the concept of ‘accelerated biological ageing’ in individuals with HIV as the ‘price to pay’ for its successful treatment [11]. There is widespread speculation as to the cause(s) of ‘accelerated biological ageing’ in individuals with HIV receiving cART. Putative causes include direct HIV effects [12,13], HIV-treatment associated effects [14,15] and behavioral or psychosocial effects [16]. The negative impact of the 'accelerated biological ageing' is speculated to be of such magnitude that many have called for a review of clinical practice and social policy in order to reduce the detrimental effects of 'accelerated biological ageing' in individuals with HIV who are on cART [17]. The idea of accelerated ageing in patients with HIV has been challenged and the observation of SNAE being observed at earlier ages has been thought to be explained by population skewing, poor life-style factors and lower socioeconomic status [18]. Socio-economic factors have been demonstrated to confound the association between HIV infection and comorbidity [19]. 

There are currently more than 30,000 Australians who have been diagnosed with HIV, with approximately 3,800 living in Queensland [20]. Moreover, half of individuals living with HIV in Queensland are aged 50 years or more [20]. Most of the current data regarding the negative impact of the ageing process come from international HIV cohorts, which are very different from the cohort of patients with HIV in Queensland. These international cohorts have been derived predominantly from North America where more than 50% of individuals with HIV in many of these research cohorts are from poorer African-American backgrounds [21,22]. Socio-economic status has been shown to be a significant predictor of outcome in HIV-infected populations [19]. On the other hand, the socialized health system in Australia provides health care access to all citizens and permanent residents [23]. These differences may limit the generalizability of these research findings to the Australian population. Furthermore, reporting of all HIV-related mortality is not mandatory in Australia therefore it is possible that deaths attributable to HIV are underestimated [24]. 

Currently there is no validated tool to measure the negative impact of the ageing process; therefore, we elected to use two potential surrogate markers of ageing: predicted mortality risk and enumeration of individual co-morbid conditions. There parameters would be expected to be higher in persons with more advanced biological age. Previous models, using CD4 count, viral load and age, used to estimate mortality in pre-cART era are inaccurate in the current era [25]. The Veterans Ageing Cohort Study (VACS) Group has developed the VACS Index to estimate the mortality risk in HIV-infected population. This index uses a score based on age, indicators of HIV disease (CD4 count and viral load) and indicators of organ-system injury such as hemoglobin, platelets, aspartate and alanine transaminase, creatinine and hepatitis C exposure (Ab +ve, PCR not included) [26]. The VACS Index has been validated in a number of different settings including the VACS cohort, hospitalized patients with HIV in the United States of America and in an European HIV infected cohort but is yet to be validated in Australia [26]. As the prevalence of individual comorbid conditions increases with age so does the likelihood that the individual patient has multiple comorbid conditions. The enumeration of these coincident comorbid conditions has been used as a surrogate measure of ageing. Guaraldi et al., demonstrated that coincident comorbid conditions (or polypathology) were more prevalent in patients with HIV compared with patients without HIV [7]. Patients with HIV were shown to have rates of polypathology that were similar to their HIV uninfected peers aged 10 years older.

The aim of the current study was to explore the negative impact of the ageing process by estimating the predicted mortality risk and quantifying co-morbidity in HIV-positive individuals on cART in Australia. Specifically, the primary objectives of the current study were: (1) to estimate the predicted mortality rates in HIV-positive Queensland males using an HIV-specific tool and compare these estimates with those using available non-HIV-specific Australian tools to assess excess of predicted mortality associated with HIV (2) to estimate the prevalence of comorbid conditions in Queensland men with HIV receiving cART (3), to examine the association between age and multiple co-morbidities in this cohort and finally (4) to examine the association between co-morbid conditions and predicted high risk of mortality within the next 5 years using the VACS Index Calculator. 

## Materials and Methods

### Ethics Statement

Access to the medical record and analysis of de-identified data was approved by The Prince Charles Hospital Human Research Ethics Committee (HREC/12/QPCH/137) and The School of Population Health Research Ethics Committee at the University of Queensland (LFK020812). It was a retrospective study using data already collected and patients' written consent was not obtained; however, a waiver from the Human Research Ethics Committees was obtained.

### Participants

The study was performed at the Brisbane Sexual Health and HIV Service, which provides healthcare to 445 men aged above 30 who have been taking cART for more than 6 months. Of these 314 were randomly selected without stratification by using the random numbers technique. Based on the 6% expected difference in mortality between Australians living with treated HIV and the Australian general population [6]; data from 310 patients were required to estimate the difference in predicted mortality with a power of 80% and a significance level of 0.05. The selection of participants was restricted to males receiving cART in order to achieve a homogeneous population, increasing the power of the study. Data extraction from the medical records was conducted during a 3 month period (September to November 2012) and charts evaluated were from patients that attended the Brisbane Sexual Health and HIV Service between January and November 2012. 

### Estimating mortality

Firstly, the probability of mortality occurring within 5 years was estimated by the online version of the VACS Index [27]. Subjects were classified as being at predicted low-risk of mortality (≤9%) or high-risk (>9%) of mortality. The threshold was set at 9% as it is the lowest risk category (0-9%) defined by the VACS Project Team [26]. The predicted risk of mortality in 5 years estimated using the VACS Index was then compared to that predicted by the Australian Bureau of Statistics (ABS) per given patient. Specific life table for Queensland men of the general population provided by the ABS was used [28]. Intrapatient differences in predicted mortality risks between the VACS Index and the ABS were analyzed by age in order to identify potential differences attributed to HIV.

### Explanatory measures

#### Demographic

Ethnicity included Aboriginal and Torres Strait Islander (ATSI), Asian, Black, White and others. Age was calculated from the date of birth reported by the patient in the medical chart and was categorized into 10-year intervals. 

#### HIV characteristics

The last recorded count of CD4 (cells/mm^3^) and viral load (copies/ml) were used . CD4 was categorized into <200, 200-349, 350-500 and >500 cells/mm^3^. Viral load was classified as undetectable (≤200 copies/ml) and detectable (>200 copies/ml).

#### Co-morbidity

Medical conditions were grouped into five organ-system clusters. 1) Metabolic disease included dyslipidemia, diabetes mellitus, and hypogonadism as reported in the medical record by the physician. 2) Cardiovascular disease (CVD) included hypertension, myocardial infarction, peripheral vascular disease, stroke and all other medical conditions defined by the American Heart Association in the International Classification of Diseases version 10 as CVD [29]. 3) Renal disease included kidney failure if the last available estimated glomerular filtration rate (eGFR) was <60ml/min and/or proteinuria if the last available urine protein/creatinine ratio was >15. 4) Cancer required a positive biopsy report of a malignant lesion. 5) Liver disease included hepatitis B (HBsAg+, anti-HBc+) and C (HCV PCR+) infection. Patients without chronic hepatitis B infection were further classified as never infected or vaccine non-responders (HBsAg-, anti-HBc-, anti-HBs-), vaccine responder (HBsAg-, anti-HBc-, anti-HBs+), cleared infection (HBsAg-, anti-HBc+, anti-HBs+). 

#### Psychiatric disorders

Psychiatric disorders included in the study were mood disorders (major depressive disorder and bipolar disorder), anxiety disorders and psychotic disorders as defined in Axis I by the Diagnostic and Statistical Manual of Mental Disorders, Fourth Edition, Text Revision (DSM-IV-TR) as recorded by the psychologist or psychiatrist [30]. Psychiatric disorders due to psychoactive substance use were not included.

#### Lifestyle

Substance use disorder included alcohol and illicit drug abuse including marijuana, amphetamines, benzodiazepines and opioids. Smoking status was categorized as never smoked, ex-smoker, and current smoker as recorded in the medical file. 

#### Poly-pathology

This was defined as having 0, 1, 2, 3 or > 3 co-morbid conditions. The rate of polypathology was analyzed by decade of age ≤ 40, 41-50, 51-60, > 60 years.

There was a concern about under-recorded comorbid conditions in the medical files; a pilot audit of 32 medical files was conducted to validate the accuracy of the documentation of comorbid conditions within the medical file. Prospective collection of co-morbid conditions was compared with retrospective collection. We only included in the study comorbid conditions that presented a substantial agreement or above (κ>0.61) [31]. 

### Statistical analyses

Differences in predicted mortality estimates using the VACS or the ABS life tables were analyzed using the student t-test. Intrapatient differences in predicted mortality risk as estimated by these alternate methods were analyzed by age. The prevalence of comorbid conditions was calculated as the proportion of people with reported comorbidities among the total sample. Chi-square test was used to assess the associations between the predicted risk of mortality and other categorical variables. Negative log-log regression was used to evaluate the association between the VACS Index (with 5 year predicted mortality risk ≤9%, coded 0/1) and the comorbid conditions. The negative log-log regression was chosen over the logistic regression since the lowest category was more probable and provided a better fit for the model [32]. The quality of the fit of the statistical model was examined using the Akaike information criterion [33]. 

Bivariate and parsimonious regression models were produced to compare the health status, psychiatric disorders and lifestyle factors reported in the predicted low-risk mortality group and the predicted high-risk mortality group. There was some overlap between the listed comorbidities and the VACS index (hepatitis C Ab +ve and renal disease through the eGFR). Therefore, the regression models were conducted without these variables. The first bivariate models, which included health status, psychiatric disorders and substance use disorders individually predicting the risk of mortality were not adjusted for any explanatory measures, whereas the second, parsimonious, model was adjusted for health status, psychiatric disorders and lifestyle factors. For the health status variables, the models were run using the organ-system clusters instead of each individual medical condition to increase the power of the findings. 

A significance level cut off of *p*<0.05 was used, and participants with missing data were excluded from the analyses. Results are presented as odds ratios (OR) and 95% confidence intervals (CI). All statistical analyses were conducted using Stata® IC, version 12.1 (Stata Corporation; College Station, TX).

## Results

Three hundred fourteen Queensland males with cART-treated HIV were included in the study. Eighty-four percent of the study population identified themselves as White and 5% as ATSI. The age range was 30 to 76 years (median 48.1 years, IQR 36.9 - 59.3 years). Thirty-nine percent of the participants were aged above 50 years. The range of CD4 count was 30 to 2000 cells/mm^3^ (median 590 cells/mm^3^, IQR 430 - 810 cells/mm^3^), only 3.2% of the sample had a CD4 count of <200cells/mm^3^ and 4.5% had a detectable viral load (Table 1).

**Table 1:** Demographics, HIV surrogate markers and medical conditions included in VACS index as proportion of total cohort and of patients with predicted high and low mortality risk as estimated by VACS index.

				Predicted risk of mortality		
		Overall		Low-risk	High-risk	Statistic
Variable		N (%)		N (%)	N (%)	p-value
Participants		314 (100)		261 (83.1)	53 (16.9)	
Ethnicity						
	Aboriginal and Torres Strait Islander	16 (5.0)		10 (62.5)	6 (37.5)	
	Asian	10 (3.3)		10 (100)	0 (0.0)	
	Black	10 (3.3)		10 (100)	0 (0.0)	
	White	265 (84.3)		218 (82.2)	47 (17.7)	
	Other	13 (4.1)		13 (100)	0 (0.0)	p=0.42
Age						
	30 - 39 years	35 (11.2)		35 (100)	0 (0.0)	
	40 - 49 years	156 (49.7)		147 (94.2)	9 (5.8)	
	50 - 59 years	80 (25.5)		64 (80)	16 (20)	
	60 - 69 years	33 (10.5)		15 (545)	18 (46)	
	≥ 70 years	10 (3.2)		0 (0.0)	10 (100)	p<0.001
CD4 count						
	<200 cells/mm^3^	10 (3.2)		3 (30)	7 (70)	
	200-349 cells/mm^3^	32 (10.2)		23 (72)	9 (28)	
	350-500 cells/mm^3^	64 (20.4)		52 (81)	12 (18)	
	>500 cells/mm^3^	208 (66.2)		183 (88)	25 (12)	p<0.001
Viral load						
	<200cpm	300 (95.5)		251 (84)	49 (16)	
	≥200cpm	14 (4.5)		10 (71)	4 (29)	p=0.23
Kidney failure (eGFR<60)		23 (7.3)		11 (48)	12 (52)	p<0.001
Hepatitis C (RNA +ve)		33 (10.5)		18 (55)	15 (45)	p<0.001

The overall mean predicted 5-year mortality risk using the VACS index was 6% and ranged from 2% to 41%. Eighty-three percent of participants were at predicted low risk of mortality (VACS index ≤9%; Table 1). In contrast, the mean predicted 5-year mortality risk using the ABS life-table was 2.1% and ranged from 0.4% to 15.7% (p<0.001) with 98.1% at low risk of mortality (≤9%). The absolute and relative mean difference in predicted life expectancy using the VACS Index and the ABS life-table were 3.9% and 2.9, respectively. Intrapatient differences in mortality risk as predicted by VACS index or ABS were constant across age groups (data not shown).

**Figure 1:**
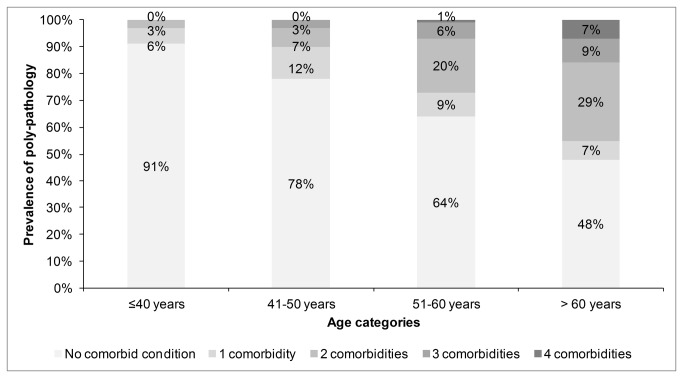
Prevalence of poly-pathology among Queensland men with cART-treated HIV, by age categories. The number of poly-pathologies or number of simultaneous co-morbidities increased with age.

**Figure 2:**
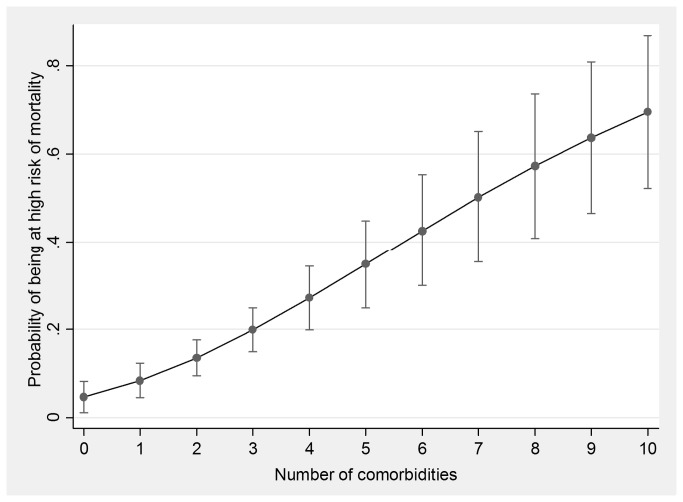
Probability and its 95% confidence interval of 5-year predicted high risk of mortality by the number of comorbidities in Queensland men with cART-treated HIV. The probability of predicted high risk mortality as estimated by the VACS index increased with the number of co-morbidities.

Metabolic diseases were the most prevalent medical comorbidities observed in this cohort (38.2%) followed by renal disease (33.1%) and CVD (23.9%; Table 2). The prevalence of psychiatric disorders was 48% with major depressive disorder and anxiety disorder being the most common conditions. The prevalence of substance abuse was high: 31% were currently taking illicit drugs, 34% were current smokers and 28% were referred to a psychologist due to their excessive alcohol intake (Table 3). The prevalence of poly-pathology increased with age, 52% of individuals aged over 60 had 1 of more co-morbid condition compared with 36%, 22% and 9% aged 51-60, 41-50 and < 40 years (Figure 1). We next examined the association between recorded comorbid conditions and 5-year predicted mortality risk as estimated by the VACS index. Firstly, 5-year predicted mortality increased with the increasing number of co-morbid conditions within the individual. Patients with 7 comorbidities had a 50% change of having a high 5-year predicted mortality risk (VACS>9%; Figure 2). This relationship existed even when co-morbidities included in the VACS index were excluded (i.e. Hepatitis C infection and kidney failure with eGFR<60).

**Table 2:** Prevalence of medical comorbidities not included in the VACS index across total cohort and as per predicted high and low risk 5 year mortality as estimated by VACS index.

				Predicted risk of mortality		
		Overall		Low risk	High risk	Statistic
Variable		N(%)		N (%)	N (%)	p-value
Participants		314 (100)		261 (83.1)	53 (16.9)	
Metabolic diseases		120 (38.2)		95 (79)	25 (21)	p=0.14
	Dyslipidemia	92 (29.3)		76 (83)	16 (17)	p=0.88
	Diabetes mellitus	22 (7.0)		15 (68)	7 (32)	p=0.05
	Hypogonadism	9 (2.9)		5 (56)	4 (44)	p=0.03
Cardiovascular diseases		75 (23.9)		52 (57)	23 (43)	p<0.001
	Hypertension	55 (17.5)		36 (69)	19 (34)	p<0.001
	Myocardial infarction	10 (3.2)		7 (70)	3 (30)	p=0.26
	Peripheral vascular disease	8 (2.5)		4 (50)	4 (50)	p=0.01
	Stroke	3 (0.9)		2 (67)	1 (33)	p=0.45
Cancer		39 (12.4)		29 (74)	10 (26)	p=0.12
Renal diseases		104 (33.1)		77 (74)	27 (26)	p=0.01
	Proteinuria	95 (30.2)		72 (76)	23 (24)	p=0.07
Hepatitis B						
	Non-responder/never infected	58 (18.5)		47 (81)	11 (19)	
	Vaccine responder	134 (42.7)		123 (92)	11 (8)	
	Cleared/Past infection	100 (31.8)		75 (75)	25 (25)	
	Currently infected	22 (7.0)		16 (73)	6 (27)	p=0.003

**Table 3:** Prevalence of psychiatric disorder as per total cohort and listed as per predicted high or low risk mortality as estimated by VACS index.

				Predicted risk of mortality		
		Overall		Low risk	High risk	Statistic
Variable		N (%)		N (%)	N (%)	p-value
Participants		314 (100)		261 (83.1)	53 (16.9)	
Psychiatric disorders						
	Psychiatric disorder	137 (43.6)		117 (85)	20 (15)	p=0.34
	Major depressive disorder	99 (31.5)		82 (83)	17 (17)	p=0.93
	Anxiety disorder	60 (19.1)		52 (87)	8 (13)	p=0.42
	Bipolar disorder	10 (3.2)		7 (70)	3 (30)	p=0.26
	Psychotic disorder	10 (3.2)		9 (90)	1 (10)	p=0.55
Substance use disorders						
	Illicit drug abuse	98 (31.2)		83 (84)	15 (16)	p=0.62
	Alcohol abuse	89 (28.3)		75 (84)	14 (16)	p=0.73
Smoking status						
	Never smoked	117 (37.3)		95 (82)	22 (18)	
	Ex-smoker	91 (29.0)		82 (90)	9 (10)	
	Smoker	106 (33.8)		84 (79)	22 (21)	p=0.10

Participants with predicted high-risk of mortality were more likely to have kidney failure and hepatitis C; however, this may be because these conditions overlapped with the calculation of the VACS Index (Table 1). Among the medical conditions not accounted for in the VACS Index, diabetes, hypogonadism, hypertension, peripheral vascular disease and hepatitis B were more likely to occur in participants with predicted high-risk of mortality (Table 2). In contrast, there were no statistically significant differences among the prevalence of psychiatric disorders, substance use disorders or smoking status between the predicted low and the high-risk mortality groups (Table 3).

Unadjusted multivariate analyses demonstrated that participants with a history of CVD had higher odds of being in the predicted high-risk mortality group than the group of participants without a history of CVD. Conversely being an ex-smoker was associated with having a lower 5 year predicted mortality risk. Responders to hepatitis B vaccination had lower odds of being in the predicted high-risk mortality group compared with participants never infected nor vaccine responders. In the parsimonious model, participants with a history of CVD were at increased odds of predicted high-risk of mortality (OR 1.69 CI 1.17-2.45) whereas ex-smokers had a predicted lower risk (OR 0.61 CI 0.41-0.92). The relationship between hepatitis B vaccination and predicted mortality risk was no longer significant (Table 4).

**Table 4:** Unadjusted and adjusted models for the association of comorbid conditions and predicted high risk mortality as estimated by VACS index.

		Unadjusted models				Adjusted model^		
			95% CI limits				95% CI limits	
Variable		OR	Lower	Upper		OR	Lower	Upper
Metabolic diseases		1.23	0.93	1.64		1.15	0.82	1.62
Cardiovascular diseases		**1.75**	**1.26**	**2.44**		**1.69**	**1.17**	**2.45**
Cancer		1.36	0.89	2.07		1.12	0.71	1.76
Hepatitis B								
	Never infected/vaccine non responder	1.00						
	Vaccine responder	**0.66**	**0.45**	**0.98**		0.73	0.48	1.11
	Cleared/Past infection	1.20	0.80	1.79		1.26	0.83	1.92
	Currently infected	1.28	0.69	2.37		1.46	0.76	2.77
Psychiatric disorders		0.87	0.66	1.15		0.87	0.63	1.20
Substance use disorders								
	Illicit drug abuse	0.93	0.69	1.25		1.07	0.72	1.58
	Alcohol abuse	0.95	0.70	1.29		1.05	0.73	1.52
Smoking status								
	Never smoked	1.00						
	Ex-smoker	0.72	0.51	1.03		**0.61**	**0.41**	**0.92**
	Smoker	1.06	0.77	1.47		1.05	0.72	1.54

*OR*: odds ratio, *CI*: confidence interval

^ Model adjusted for metabolic disease, cardiovascular disease, cancer, hepatitis B, psychiatric disorders, substance use disorders and smoking status.

NOTE. Statistically significant ORs are emboldened.

## Discussion

The mean predicted 5-year mortality for Queensland males with cART-treated HIV using the VACS Index (6.0%) is higher when compared with the predicted mortality risk using ABS life table data for the same patients (2.1%) [28]. This suggests that Queensland males with cART-treated HIV present almost a threefold increased probability of death within the next 5 years than Queenslanders free of HIV infection of the same gender and age. In absolute terms, a 65 year old male has a predicted 5-year mortality risk which is 3.9% higher than a 50 year old male using the ABS life table data. The findings of increased predicted mortality in HIV infected men on treatment is consistent with other Australian reports which demonstrated that the standardized mortality rate (SMR) of a cohort of very long term infected HIV infected patients was 3.5 [5,6]. In these previous studies not all patients were taking cART and report mortality across a period from 1999-2004 when mortality rates for patients with HIV may have been expected to be higher than current rates. Our findings may therefore suggest that this previously documented excess mortality risk persists within a contemporary treated cohort. Alternatively, the VACS Index may over-estimate the predicted mortality risk in Australian men on cART. It is interesting to speculate that social, behavioral and health care access issues may underscore this apparent paradox. These issues are unable to be resolved with the currently available data until the VACS Index is validated within the Australian context.

The prevalence of comorbid conditions in males with cART-treated HIV in this cohort was greater than that observed in the Australian general population. For instance, diabetes mellitus (7.0% among Queensland males with cART-treated HIV vs. 5.0% among the Australian general population), hypertension (17.5% vs. 11.0%) and renal diseases (33.1% vs. 0.8%) were more prevalent in Queensland males with cART-treated HIV than in the Australian general population of the same gender and age range according to the ABS (2012)[34]. The marked increase in renal disease in patients with treated HIV may be related to antiretroviral therapy associated renal dysfunction [35]. We have previously demonstrated that 27% of patients taking tenofovir for more than one year had proteinuria; in addition, the proteinuria was reversible in 92% of the patients after tenofovir was ceased [36]. Thus, the high prevalence of renal impairment observed in this study might be transient and associated with the cART administration rather than established kidney disease due to hypertension or diabetes mellitus. Proteinuria has been demonstrated to predict mortality in patients with treated HIV [37]. Additionally, the prevalence of hepatitis C (10.5% vs. 0.05%) [20], hepatitis B (7.0% vs. 0.03%) [20] and psychiatric disorders (48.7% vs. 20%) [38] were higher in our study population. Illicit drug abuse (31.2% vs. 15.3%) [38], alcohol abuse prevalence (28.3% vs. 12.0%) [38] and rates of smoking (33.8% vs. 23.0%) [39] were also found to be higher in Queensland males with cART-treated HIV than that observed in age- and gender-matched persons from the general Australian population. Similar increased rates of co-morbidity in other cohorts of HIV infected Australians have been reported [40,41].

We next enumerated individual comorbid conditions within the same patient. This ranged from zero to seven (Figure 1). Older patients were more likely to have multiple co-morbidities. This pattern, previously described by Guaraldi et al., had been used as a surrogate marker of biological ageing in a cohort of HIV-infected Italians [7]. However differences in the types of co-morbid conditions included in that study and our study limits comparisons between the studies being made. We also examined the association between multiple comorbidities and the VACS Index. We found that the VACS Index increased with increasing number of comorbidities within the same individual patient (Figure 2). This relationship held even after accounting for age, hepatitis C exposure and eGFR (factors used in computing the VACS Index). 

Secondly, patients with a history of CVD had higher odds of being at predicted high-risk of mortality (VACS>9%) in multivariate analyses. This result is supported by a previous report [42] which demonstrated that patients with risk factors for cardiovascular disease had higher VACS Index, despite these factors not being included in the computation of the score. These findings are consistent with the demonstration that CVD is a leading cause of death among people living with HIV [43]. We also found that patients who ceased smoking had a lower predicted risk of mortality than current smokers or those who never smoked in our study. This finding might be confounded because of the additional information ex-smokers receive about healthy life style; additionally, they are more likely to seek medical consultation than patients that never smoked [44]. Smoking cessation has been associated with reduced risk of subsequent CVD in patients with HIV [45]. 

Our study is limited by a number of factors. Firstly, pre-existing data were utilized in the current analyses, and without a prospective design causality is difficult to establish. We were able to be more confident of our findings by only including co-morbidities known to be well recorded in the medical record. Secondly, access to actual mortality data of Queensland males with cART-treated HIV was limited. Thus, the VACS Index was used to estimate the predicted mortality in Queensland males with cART-treated HIV. This score estimates the risk of mortality more accurately among people aged 50 years and above and those with an undetectable viral load than indexes restricted to CD4 count and viral load [26,46]. The VACs Index is yet to be validated in Australia so it may be inaccurate. It may overestimate the predicted risk of mortality in Australian patients. While sociobehavioral, socioeconomic and access to health care differences may explain the differences between Australian and United States outcomes, until the VACS Index is validated in Australia the findings although provocative will remain speculative. Socioeconomic factors have been shown to influence clinical outcomes in contemporary HIV treated populations [16,43]. However, we were unable to account for socioeconomic status factors in our cohort as this data was not systematically collected. Thirdly, a limited sample size was obtained and therefore reduced the power. To correct for this the sample was restricted to a homogeneous group of participants that included males above 30 years and receiving cART. Eighty-five to ninety percent of Queenslanders living with HIV are males over the age of 30 and on ART and so would be represented by the cohort studied [47]. Therefore, generalizability of our findings to women (and men not on antiretroviral therapy) is limited. The lack of an appropriate control group imposes an additional limitation, the inability to adjust for possible confounders such as lifestyle factors and number of comorbidities among HIV-negative men might have affected the associations found. 

Our findings suggest that HIV infected men on cART are negatively impacted by an increased burden of biological ageing as indicated by an apparent increased predicted risk of mortality over the next five years and an increased prevalence of comorbidities compared with the Australian general population. The demonstration that established CVD was strongly associated with this increased predicted risk of mortality and that ex-smokers had a lower overall predicted mortality risk support efforts to decrease cardiovascular risk in patients with HIV. While these findings may support the notion that the burden of biological ageing is increased in HIV-infected Australian men on cART until the VACS Index is validated in the Australian context the findings while provocative remain speculative.
